# A novel coil array for combined TMS/fMRI experiments at 3 T

**DOI:** 10.1002/mrm.25535

**Published:** 2014-11-24

**Authors:** Lucia I. Navarro de Lara, Christian Windischberger, Andre Kuehne, Michael Woletz, Jürgen Sieg, Sven Bestmann, Nikolaus Weiskopf, Bernhard Strasser, Ewald Moser, Elmar Laistler

**Affiliations:** ^1^ Center for Medical Physics and Biomedical Engineering, Medical University of ViennaViennaAustria; ^2^ MR Centre of Excellence, Medical University of ViennaViennaAustria; ^3^ Department of Biomedical Imaging and Image‐Guided TherapyMedical University of ViennaViennaAustria; ^4^ Wellcome Trust Centre for Neuroimaging, University College LondonLondonUnited Kingdom; ^5^ Sobell Department of Motor Neuroscience and Movement Disorders, UCL Institute of Neurology, University College LondonLondonUnited Kingdom

**Keywords:** TMS, fMRI, coil array, parallel imaging, concurrent TMS/fMRI

## Abstract

**Purpose:**

To overcome current limitations in combined transcranial magnetic stimulation (TMS) and functional magnetic resonance imaging (fMRI) studies by employing a dedicated coil array design for 3 Tesla.

**Methods:**

The state‐of‐the‐art setup for concurrent TMS/fMRI is to use a large birdcage head coil, with the TMS between the subject's head and the MR coil. This setup has drawbacks in sensitivity, positioning, and available imaging techniques. In this study, an ultraslim 7‐channel receive‐only coil array for 3 T, which can be placed between the subject's head and the TMS, is presented. Interactions between the devices are investigated and the performance of the new setup is evaluated in comparison to the state‐of‐the‐art setup.

**Results:**

MR sensitivity obtained at the depth of the TMS stimulation is increased by a factor of five. Parallel imaging with an acceleration factor of two is feasible with low g‐factors. Possible interactions between TMS and the novel hardware were investigated and were found negligible.

**Conclusion:**

The novel coil array is safe, strongly improves signal‐to‐noise ratio in concurrent TMS/fMRI experiments, enables parallel imaging, and allows for flexible positioning of the TMS on the head while ensuring efficient TMS stimulation due to its ultraslim design. Magn Reson Med 74:1492–1501, 2015. © 2014 The Authors. Magnetic Resonance in Medicine published by Wiley Periodicals, Inc. on behalf of International Society for Magnetic Resonance in Medicine.

## INTRODUCTION

Functional magnetic resonance imaging (fMRI) is one of the most important brain‐activity mapping techniques and is widely used in the neurosciences, including psychology, psychiatry, and neurology. In fMRI, brain activity is assessed indirectly by detecting MR signal changes from alterations in blood oxygen levels and blood flow during neural activity. Despite its ample usage, this indirect contrast mechanism is also the reason for the strongest caveats against fMRI. Combining fMRI with transcranial magnetic stimulation (TMS), which allows for direct interaction with the neuronal substrate, can thus prove a desirable approach to overcome these limitations.

TMS was introduced by Barker et al. 1 as a new noninvasive, painless neurological tool. For almost 30 years, TMS has been utilized in modern medicine. It is not only a powerful diagnostic tool 2, 3, 4, 5, 6, 7 but has also become an important technique for cognitive neuroscience. One major advantage of TMS is that it can be used to interact with the function of single brain regions at well‐defined time points. As such, it can reveal causality relations between different areas within a neural network. In addition, repetitive TMS (rTMS) has been used as a therapeutic device in many neurological and psychiatric conditions, especially in the treatment of major depression 8, 9, 10, 11.

Fifteen years ago, Bohning et al. 12 showed the feasibility of combining TMS with fMRI. The purpose was to acquire functional brain images immediately after cortical stimulation in order to combine the benefits of TMS (i.e., well‐defined temporal interaction with neurons) with the benefits of fMRI (i.e., high spatial resolution). The current state‐of‐the‐art setup for these experiments comprises a large‐volume head coil, usually a birdcage coil, with the MR‐compatible TMS coil being mounted inside that birdcage coil. It was applied in a multitude of experiments studying local and network interactions 13, 14, 15, 16, 17, 18, 19, 20, 21, 22, 23, 24, 25, 26, 27, 28, 29, 30, 31. For these experiments, methods were developed to avoid artifacts 32, 33, signal loss during the acquisition 34, and to find the most suitable rTMS protocols 35 or cortical targets 36. Reviews of the applications and methodology in concurrent TMS/fMRI can be found in the literature 37, 38, 39, 40, 41.

In concurrent TMS/fMRI studies, the state‐of‐the‐art setup has many disadvantages. The positioning of the TMS coil on the subject's head has always been a challenge because the limited space inside the birdcage coil impedes TMS positioning. Although new coil positioning methods have been developed 42, 43 with integrated software and graphical user interface to facilitate accurate placement of the TMS coil inside a scanner, there are still some brain areas that cannot be stimulated in the scanner, or for which positioning remains very difficult and uncomfortable for the subjects.

Another technical limitation of the birdcage coil setup is that the achieved signal‐to‐noise ratio (SNR) is very limited compared to state‐of‐the‐art multichannel head coils being used nowadays in fMRI studies 44. Unfortunately, the large birdcage coil cannot simply be replaced by a multichannel coil; these coils are usually more form‐fitted to the head, and there would be no space left to position the TMS between the head and the MR coil. On the other hand, the coil housings are too thick to place the coil between the head and the TMS, thus preventing efficient stimulation with the TMS coil due to the increased distance.

Also, it is not possible to implement parallel imaging 45, 46 using birdcage coils because multiple receive channels are required for these techniques. To date, TMS/fMRI studies could not benefit from the significant reduction in scan time by parallel imaging, and various recent developments in fMRI such as multiband sequences 47 could not be used.

**Figure 1:**
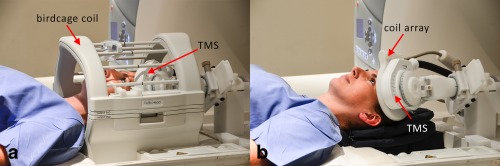
(a) Conventional setup for concurrent TMS/fMRI experiments. The TMS coil is placed between the birdcage coil and the head of the subject. (b) New setup using the novel coil array. The MR coil array is placed between TMS and the head of the subject.

To overcome these issues, a dedicated, multichannel receive MR coil array for concurrent TMS/fMRI experiments is presented, providing a new way of conducting such experiments. The novelty consists in the design of a very slim coil array that can be easily attached to the TMS and placed between the subject's head and the TMS device (see Figure 1). Due to its thin design, TMS stimulation amplitudes are acceptable. The spherically curved housing of the MR coil fits various parts of the head and should allow for positioning on areas that are not accessible with the state‐of‐the‐art setup.

Although TMS devices have been previously used in combination with birdcage head coils, interactions between TMS and coil array need to be evaluated for the proposed arrangement to ensure safe use and avoid disturbance in the function of either of the two devices. Especially due to their proximity and the high magnetic flux density routinely employed in TMS experiments (about 1 T at frequencies of about 1–10 kHz), possible interactions are carefully studied in this work.

## METHODS

### Coil Design and Construction

**Figure 2:**
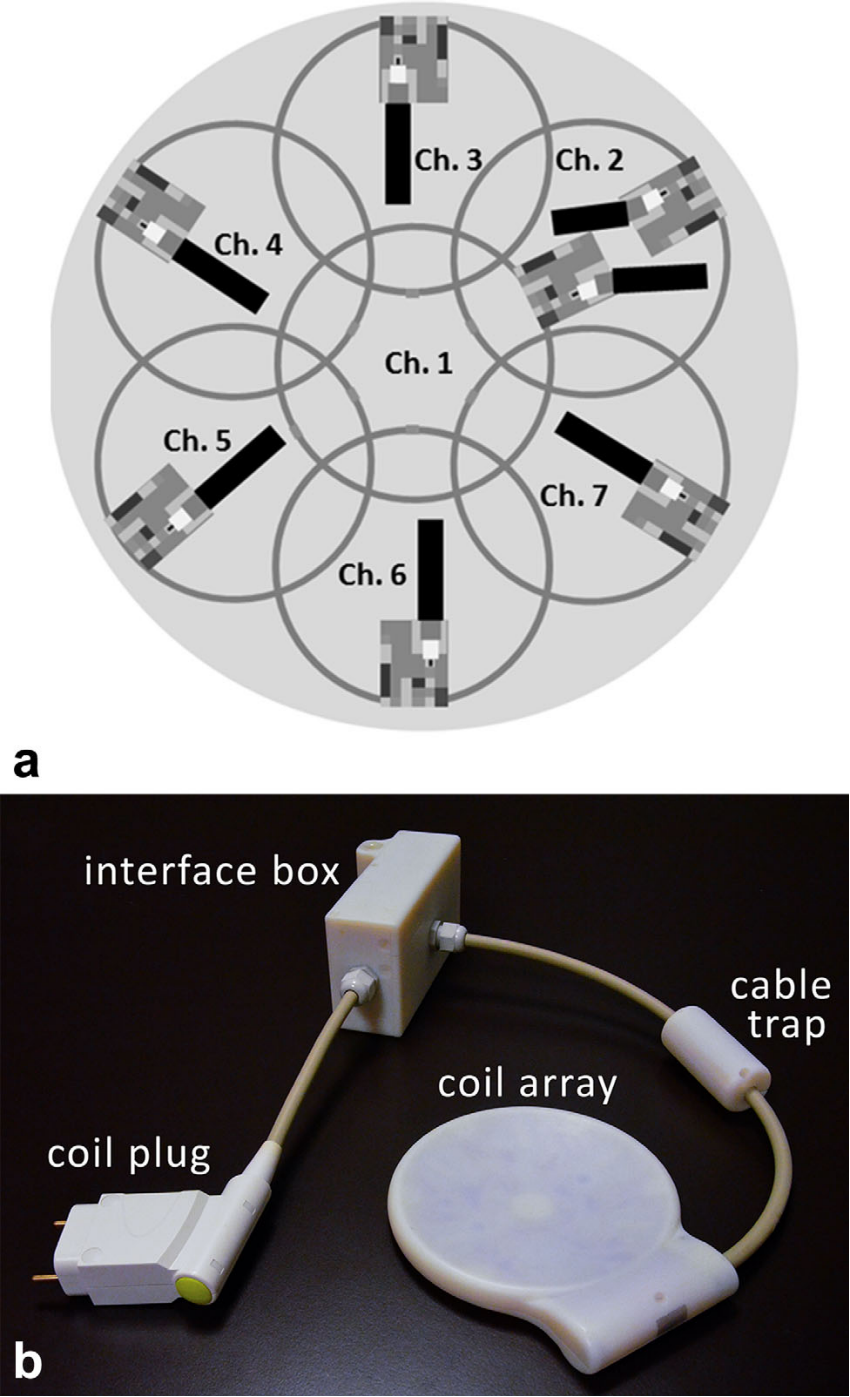
(a) Arrangement of the single surface loops of the coil array developed for simultaneous TMS/fMRI studies. (b) Photograph of the novel MR head coil array for concurrent TMS/fMRI experiments, together with its interfacing hardware.

For the design of the dedicated MR device to work simultaneously with TMS, basic surface loops were chosen due to their intrinsically high SNR. The field of view of the MR coil should be centered on the stimulation spot, and the target depth for cortical studies is in the range of 5 to 7.5 cm. With these requirements, an overlap‐decoupled, hexagonally arranged 7‐channel phased array was designed (see Figure 2a). A diameter of 6 cm was chosen for each loop, which is the optimal choice to obtain an optimal SNR for the desired target depth 48.

**Figure 3:**
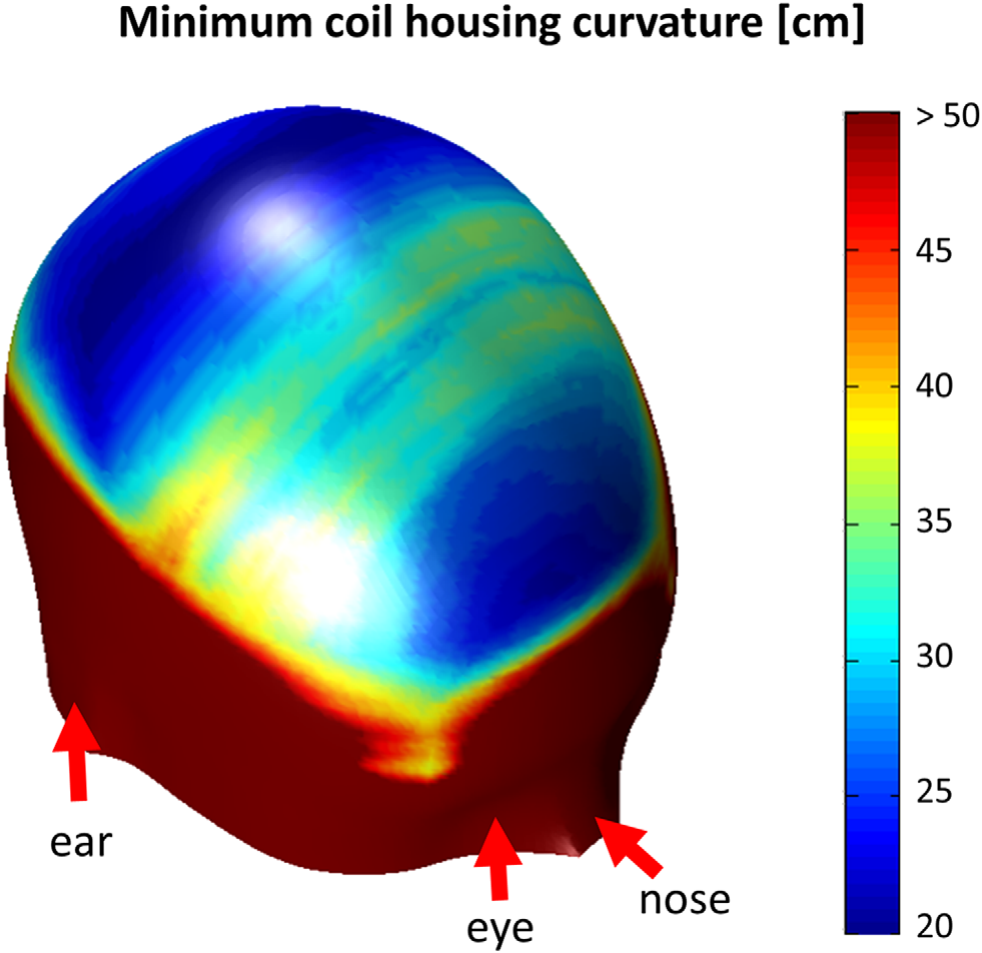
Local minimum coil housing curvature mapped onto the surface of an MNI space head model (see Methods section of article for detailed description). For regions of the head, where its curvature is strong (i.e., in frontal areas and on the parietal bone), low values (blue) of coil curvature around 20‐ to 25‐cm in diameter could be used. For accessing the flatter areas of the head (e.g., somatosensory and motor areas), a flatter coil housing also is needed (30–40 cm; green/yellow areas). A curvature of 45 cm was needed for the coil housing used in this study, which makes most parts of the brain accessible to TMS stimulation with the MR coil array in place.

The head coil is constructed on a thin plastic housing with a spherical curvature, built using a three‐dimensional (3D) printer (Objet Eden 350V; Stratasys, Eden Prairie, MN, USA). The curvature should be suitable for most head shapes while keeping the coil elements close to the skull for sensitivity. To obtain the minimum required curvature diameter, a surface mesh model of a head in MNI‐space was obtained from a voxel image template (single_subj_T1.nii, SPM12b toolbox, http://www.fil.ion.ucl.ac.uk/spm/) using MATLAB Version 7.8.0 R2009a (The MathWorks, Natick, MA, USA). For each triangular element of the surface mesh, spherical caps with their respective central points located at the head surface were created. Their curvature diameters were varied between 20 and 50 cm in 1‐cm steps, and their lateral extension was adjusted for each curvature diameter to accommodate the whole coil array on the cap. At each position of the head surface, the minimum curvature diameter for which the cap did not collide with the head was stored. According to the result displayed in Figure 3, a diameter of 45 cm was chosen for the coil housing, suitable for a large variety of head shapes and target regions on the head. The fixation mechanism of the TMS device with the MR coil was designed to allow existing TMS coil‐positioning systems to be used (see Figure 1).

The basic coil elements were constructed using 1‐mm‐diameter insulated copper wire. Each loop was segmented symmetrically into two parts by a capacitor. Nonmagnetic capacitors (CHB series, Temex Ceramics, Pessac, France) were employed in the loop and on a printed circuit board containing the matching and decoupling networks. With the coil array positioned on the head, each surface loop of the array was tuned to 123.25 MHz and matched to 50 Ω. On the printed circuit boards, a triple protection system was implemented to avoid possible induced high currents during the transmission part of the MR acquisition. These three mechanisms are: a series fuse with 315 mA nominal current rating in series with the loop, an active decoupling network, and a passive decoupling network. For the active and passive detuning circuit, toroidal inductors were wound, constructed from 0.5‐mm‐diameter insulated copper wire. For active detuning, a PIN diode is used (DH 80106, Temex Ceramics). This diode is biased through a radio frequency (RF) choke (1812CS; Coilcraft, Cumbermauld, UK). The passive detuning circuits consist of a direct current (DC) block capacitor (CHB series, Temex Ceramics), RF fast diodes (UM9989; Microsemi, Aliso Viejo, CA, USA) and an RF choke inductor to short the switching currents, minimizing noise created by the fast diodes with small DC currents.

Nearest‐neighbor coil elements were decoupled by the classical overlap method. In order to minimize coupling between nonoverlapped elements, preamplifier decoupling was implemented as a second‐order matching network 49 for each array element. The structure A‐2 proposed in Reykowski et al. 49 was selected to minimize coupling between the inductor used for preamplifier decoupling and the inductor used for detuning.

The low‐noise preamplifiers (noise figure = 0.5 dB, gain = 27.2 ± 0.2 dB) (Siemens Healthcare, Erlangen, Germany) were placed in a separate interface box. This keeps the coil very thin, resulting in a thickness of 4.5 mm in the center of the coil, and also creates sufficient distance between the TMS coil and the preamplifier electronics.

In order to suppress common mode currents induced on the cable shields during transmission, floating cable traps 50 were placed on the coaxial cables that connect the single elements to the interface box. The ensemble of the coil array, including its interface box and connector plug, is shown in Figure 2b.

### Bench Measurements

Measurements of the scattering parameters (S‐parameters) were performed using a vector network analyzer (E5071C; Agilent Technologies, Santa Clara, CA). A custom‐made test rig was constructed to provide the required 10‐V power supply for the preamplifiers and enable manual switching of the bias lines from current (100 mA) to reverse bias (−30 V).

The loaded quality factors (Q_L_) were determined by the −3 dB bandwidth method from an S_ii_ measurement. The unloaded quality factors (Q_U_) were determined by the −3 dB bandwidth method from an S_ij_ measurement with two overlap‐decoupled (≈ −80 dB) pickup loops.

Tuning and matching of the array elements was done by measuring the reflection coefficient S_ii_ for each channel loaded by a human head, with the remaining elements connected to the respective preamplifiers. Preamplifier decoupling was tested according to the method proposed by Reykowski et al. 49.

The loaded coupling matrix of the array coil was measured as direct S_ij_ measurement between the selected channels, with all other elements being terminated with 50 Ω.

### Measurements of the MR Coil Interaction With the TMS System

Interactions of the novel hardware with the TMS system placed on top of it were evaluated to ensure that both devices work properly and safely in combination.

The main concern was that currents induced in the MR coil during TMS stimulation might damage hardware or even harm the patient. To obtain a first estimate of these currents, the maximum induced voltage in a coil element by the TMS pulse was computed using Faraday's law of induction on a wire loop coil of the same size and shape as the array elements.

On the bench, the induced voltage on a surface loop during a TMS pulse with maximal amplitude was measured with an oscilloscope with high input impedance (TDS3052; Tektronix, Berkshire, UK). To avoid voltage division, the surface loop was built without segmenting capacitors. The TMS was placed in a way to share maximal magnetic flux with the loop and thus achieve maximal induction. Using one of the segmented coil array elements in the same configuration, the voltage at the preamplifier input was also measured.

For a more detailed analysis of induced currents and voltages, one coil element including decoupling and detuning circuits was simulated using a circuit simulation software package (Advanced Design System, Agilent Technologies). The previously measured induced voltage in the loop was used in the simulation to compute the current in the loop and the maximum voltage at the preamplifier input during TMS stimulation.

A second concern, the efficiency loss of the TMS magnetic field due to increased distance between patient and TMS coil was investigated by quantitative mapping of the TMS magnetic fields using MR phase images 51. Because standard TMS coil currents are too high for this approach, a dedicated low‐current pulse generator supplied by the TMS manufacturer (MagVenture, Farum, Denmark) was used to deliver 1‐ms block pulses to the TMS system in the range of 0 to 400 mA. Measurements were performed on a clinical 3 T MR scanner (Magnetom Trio [Tim System], Siemens Healthcare), with the TMS device (MRi‐B91; MagVenture, Farum, Denkmark) attached to a spherical phantom (D165, Siemens Healthcare) with and without the coil array placed between the phantom and the TMS device. Data were acquired with the body coil for both measurements. To verify the results in vivo, the change in motor threshold on a healthy male volunteer (41 years old) was measured with and without the MR coil array between the TMS coil and the head. In both cases, the TMS coil was positioned as closely to the head as possible.

Thirdly, the influence of the TMS on the resonance frequency of each element of the MR coil array was investigated. The respective resonance frequency shifts were measured on the network analyzer.

### MRI Data Acquisition and Reconstruction

To verify the effective isolation of the receive‐only coil array from the body coil during transmit, B_1_^+^ field maps (repetition time [TR]/echo time [TE] = 1000 ms/14 ms, flip angle = 25°, 22 slices, 5‐mm slice thickness, field of view [FOV] = 200 × 200 mm^2^, matrix size [MA] = 128 × 128, bandwidth [BW] = 206 Hz/pixel), were acquired with the body coil. The field maps were compared to those obtained without the coil array present. The relative difference between the two measurements was calculated.

In order to compare the conventional setup using the scanner manufacturer's birdcage coil (see Figure 1a) to the new setup with the developed coil array (see Figure 1b), measurements were performed on a healthy female subject (39 years old) after written informed consent. The new MR device was positioned under the head, with the TMS coil centered at the occipital pole. This allowed for acquiring images of the occipital lobe. The sequence used for the SNR maps was a 3D gradient recalled echo; the parameters were TR/TE = 50 ms/3.03 ms, flip angle = 15°, 120 slices, 1.5 mm slice thickness, FOV = 192 × 192 × 180, MA = 128 × 128, and BW = 501 Hz/pixel. To obtain noise covariance information 52, a free induction decay sequence (TR/TE = 240 ms/2.87 ms, BW = 10 kHz, without RF excitation) was used to acquire noise data. To avoid long measurement times, 50 averages were acquired and then concatenated to a longer time series in postprocessing. To calculate the SNR maps, raw data sets were prewhitened and reconstructed using the sum of squares of the magnitude images based on the method by Kellman et. al. 52.

A magnetization‐prepared rapid acquisition gradient echo (MPRAGE) sequence was also performed with the following parameters: TR/TE/inversion time (TI) = 2300 ms/4.14 ms/900 ms, flip angle 9°, 160 slices, 1.1 mm slice thickness, FOV = 265 × 283 mm^2^, MA = 230 × 256, and BW = 238 Hz/pixel.

To assess the parallel imaging performance of the array, g‐factor maps were computed. For the calculation of these maps, measurements were done on the spherical phantom, providing a coil load similar to the human head. Coronal and sagittal gradient echo images were acquired with TR/TE = 500 ms/2.87 ms, flip angle 25°, 30 slices, 3 mm slice thickness, FOV = 192 × 192 mm^2^, MA = 192 × 192, and BW = 501 Hz/pixel. The phase‐encoding direction was foot to head. Noise data was also acquired with the sequence described above. The original raw data were decimated with acceleration factors R = 2 and R = 3. Then, using the pseudo‐multiple replica method 53 and the generalized autocalibrating partially parallel acquisitions (GRAPPA) reconstruction algorithm 45, g‐factor maps were calculated using MATLAB. The obtained maps were smoothed with a 3 × 3 pixel mean filter.

### TMS/fMRI Experiment

To validate the feasibility of the new setup, a concurrent TMS/fMRI experiment on the motor cortex was designed. The TMS‐coil used for the experiment was an MR‐compatible coil (MRi‐B91; MagVenture, Farum, Denmark).

The experiment was conducted on a healthy right‐handed male volunteer (41 years old) after written informed consent, placing the TMS coil fixated to the coil array over the primary left motor cortex. TMS coil position and active motor threshold were validated by applying single TMS pulses. The fixated TMS coil with the coil array was placed using the vendor's positioning system (MRi Coil Holder; MagVenture). Head movement was restricted by foam‐padded cushions, and the subject wore earplugs throughout the experiment.

The experiment was performed in a 3 T scanner. Functional images were acquired using a 2D single‐shot echo‐planar imaging (EPI) sequence with TR/TE = 2000 ms/33 ms, flip angle 60°, 10 slices, 3 mm slice thickness, 2 × 2 mm^2^ in‐plane resolution, MA = 92 × 92, and BW = 1598 Hz/pixel. The acquired slices were aligned in parallel to the TMS coil and fully covered the primary motor cortex. Anatomical MPRAGE images were also acquired (TR/TE/TI = 2300 ms/4.32 ms/900 ms, flip angle 9°, 160 slices, 1.25‐mm slice thickness, FOV = 202 × 216 mm^2^, MA = 240 × 256, and BW = 238 Hz/pixel).

**Figure 4:**
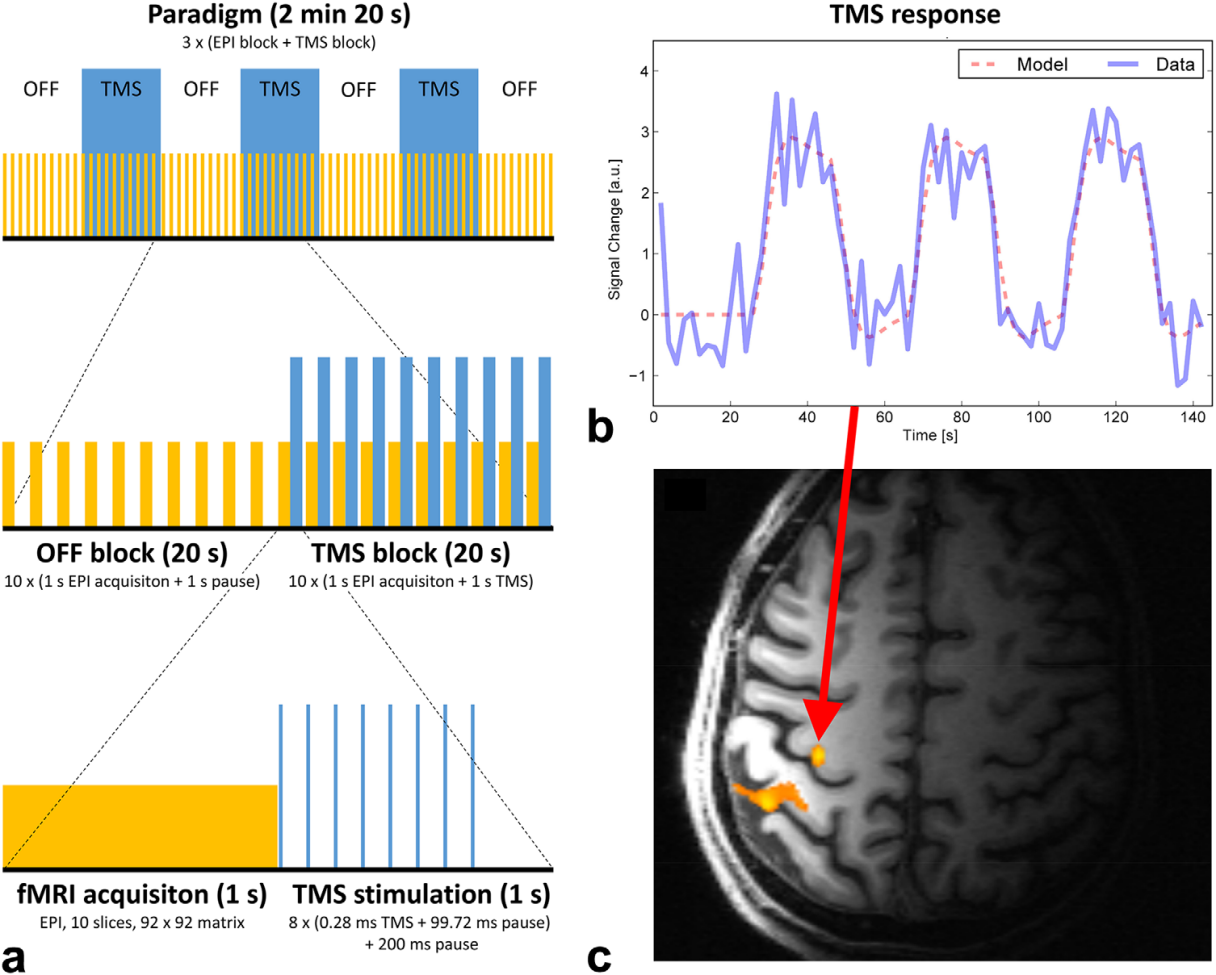
TMS/fMRI experiment. (a) The paradigm used for showing the feasibility of the new hardware for combined TMS and fMRI. Yellow blocks depict fMRI data collection. Blue blocks correspond to TMS stimulation periods. (b) The TMS stimulation time course convolved with SPM's canonical hemodynamic response function is shown together with the signal time course from the voxel with highest activation. c) The activation map (*p* < 0.05; family wise error corrected) overlaid on the corresponding T1‐MPRAGE anatomical reference image. The maximum of activation was found in the hand area of the primary motor cortex.

The stimulation protocol is depicted in Figure 4a. The paradigm comprised three alternating “off” and “TMS” blocks, as well as an additional “off” block at the end; that is, a total of three TMS blocks were applied (see the top row of Figure 4a). Within each TR of 2 s, imaging was restricted to the first half, whereas TMS was performed in the second half of TR to avoid interferences. Each TMS block contained 10 stimulation trains, each train consisting of eight biphasic 280‐μs‐long TMS pulses at a frequency of 10 Hz, as shown in the bottom row of Figure 4a. Via the serial port of the TMS stimulator, an in‐house written MATLAB script controlled the TMS timing with respect to an optical trigger signal from the MR scanner at the beginning of each volume acquisition. Stimulation intensity was set at 110% of the individual's active motor threshold. This threshold was defined as the minimal percentage of the stimulator output that produced a clear visible twitch of the right index finger in four out of eight pulses during slight voluntary contraction 54. The stimulation protocol was in line with established safety guidelines 41.

Data analysis was performed using the SPM12b toolbox. Preprocessing of fMRI acquisitions included slice timing correction 55, motion correction, and smoothing with a 3‐mm full width at half maximum isotropic Gaussian kernel. The preprocessed volume time courses were analyzed using the general lineal model (GLM), as implemented in SPM. The time course of the TMS blocks, as shown in blue in Figure 4a (top row), was convolved with SPM's canonical hemodynamic response function and used as task regressor in the design matrix. A standard single‐subject analysis was performed by applying a *t*‐test to the GLM parameter estimates.

## RESULTS

### MR Coil Properties

The unloaded quality factor Q_U_ averaged over all channels was 121.7 ± 8.3. When loaded by a head, the loaded quality factor Q_L_ was 51.7 ± 5. The resulting ratio Q_U_/Q_L_ is 2.4 ± 0.2, indicating that the total noise is sample‐dominated.

**Figure 5:**
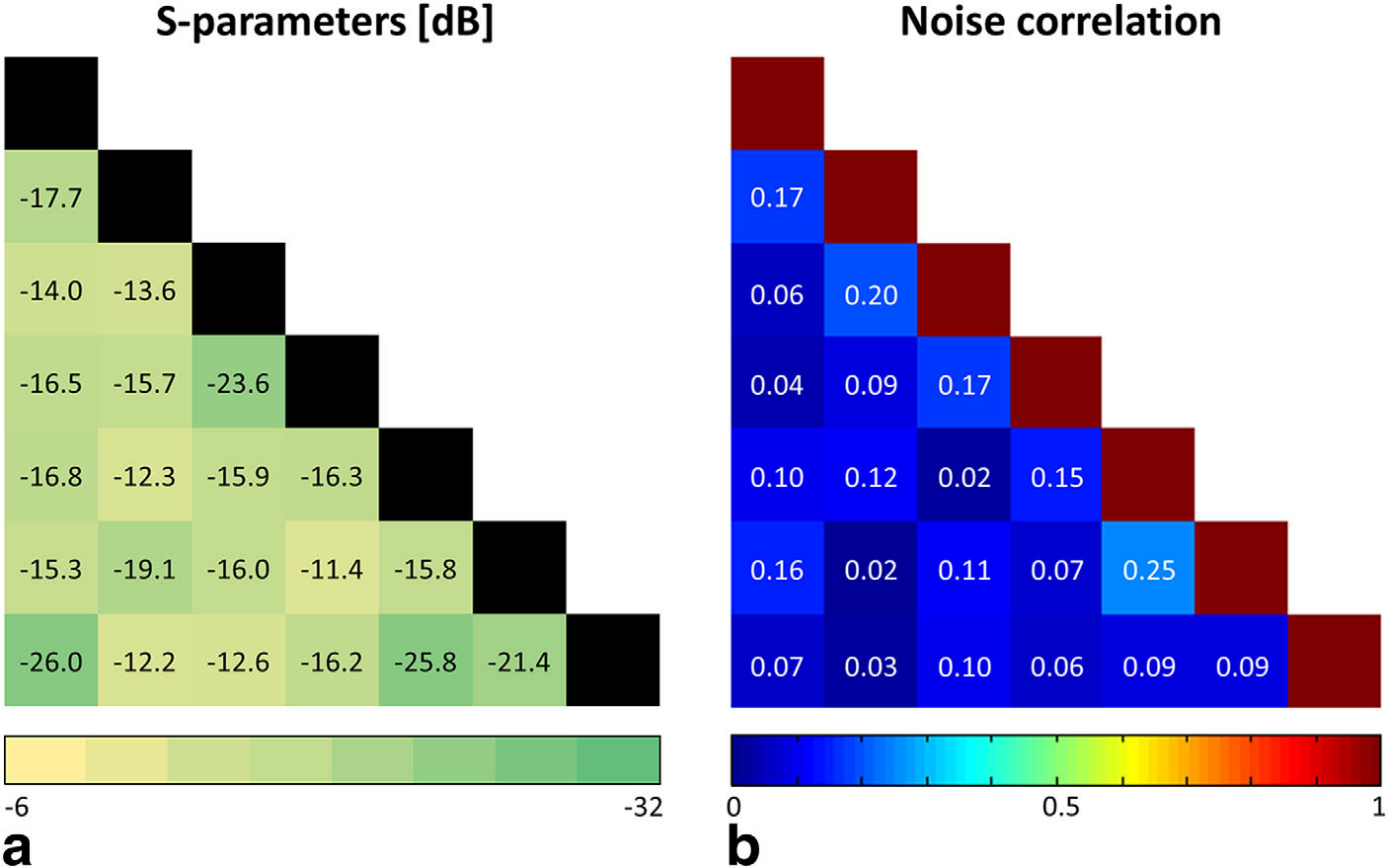
S‐parameter and noise correlation matrices. (a) S_ij_ measurements for each channel pair; all other channels terminated with 50 Ω. (b) Noise correlation matrix for the coil array loaded by a spherical head phantom and the TMS device attached.

The active detuning efficiency was measured to be better than 40 dB for all elements. These results were verified in the MR scanner, with variations of the flip angle in the B_1_^+^ field maps below 5% (not shown). The results of the S‐parameter measurements are presented in Figure 5a. The range of coupling between overlapping neighboring elements (see Figure 2a for the channel arrangement) varied from −12.2 dB to −26 dB, with an average of −16.6 dB. Non‐neighboring element coupling ranged from −11.4 dB to −25.8 dB, with an average of −15.3 dB. Preamplifier decoupling added another −15 dB.

Noise correlation values ranged from 1.6% to 25.2%, with an average of 10.3% for off‐diagonal elements (Fig. 5b). Channel 1 is placed in the center of the coil and overlaps with all other channels. Therefore, the sensitivity patterns overlap as well, and partially the same noise signal is acquired. Hence, the corresponding average correlation of overlapping channels was 12%, in contrast to an average of 8% for nonoverlapping channels.

### Interactions Between MR Coil and TMS System

**Figure 6:**
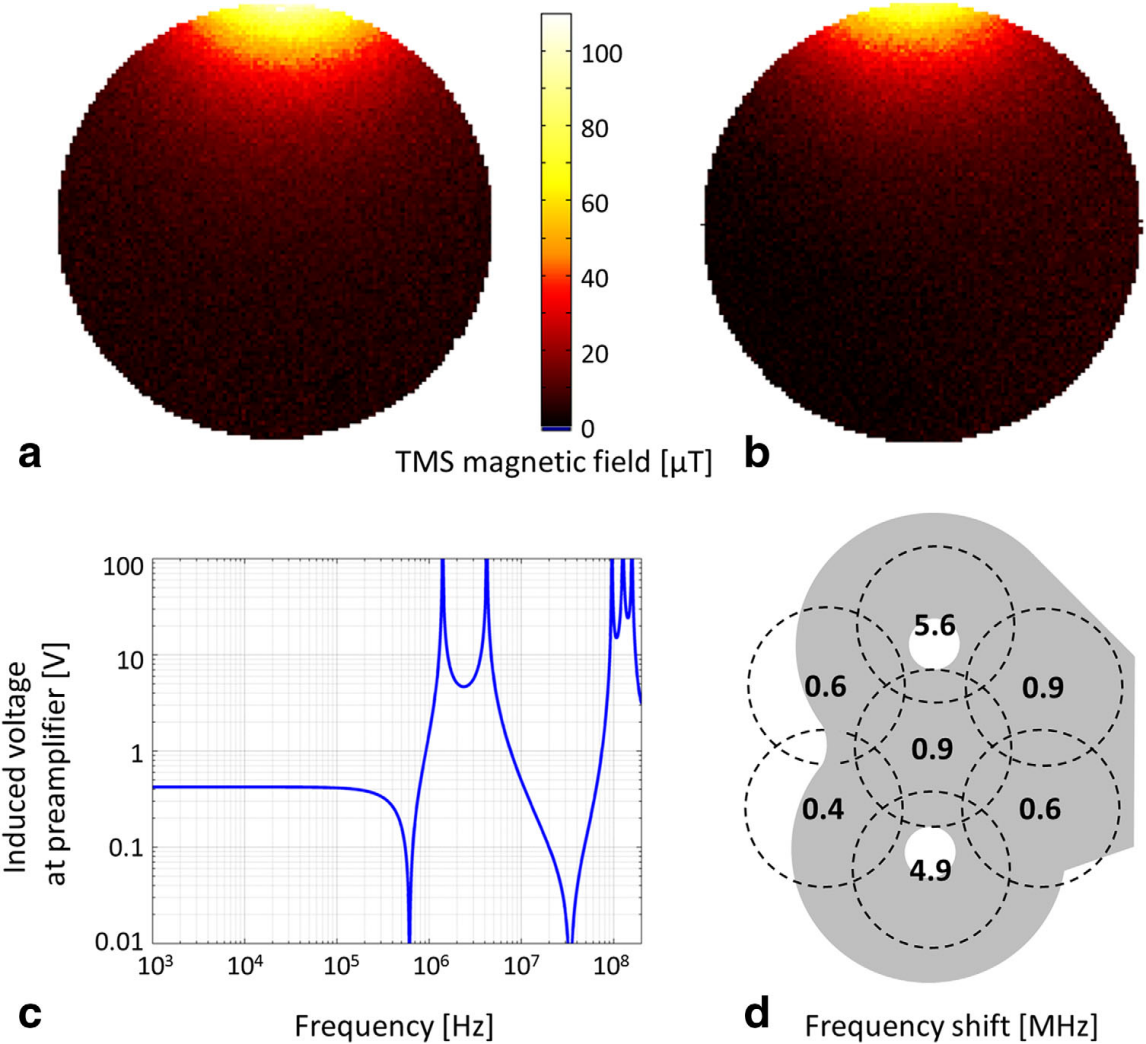
Interactions between MR coil array and TMS system. (a) and (b) TMS magnetic field map of a tangential component (x) with 150‐mA TMS coil current. Measured data (a) without and (b) with the MR coil between TMS and phantom. (c) Calculated voltage induced at the preamplifier input as a function of frequency. Peaks between 1 and 10 MHz can be attributed to resonances of RF chokes and DC‐block capacitances. Voltages below 100 kHz are constant at 0.4 V. (d) Shifts in resonance frequency for each channel of the coil.

The tangential component B_x_ of the magnetic field created by the TMS device is shown in Figures 6a,b. TMS efficiency loss with the array between the TMS and the phantom was measured as ≈ 16% and 14% at 1.5 cm and 2.5 cm depth in the phantom, respectively. The motor threshold of the volunteer changed from 66% to 84% of the maximum TMS power setting when the MR coil array was placed between TMS and the volunteer's head.

The theoretical voltage, as calculated from Faraday's law, induced in a single loop by a 1 T amplitude TMS pulse at 3 kHz, was 53 V. Measurements using the simple wire loop and the TMS coil at 100% of its current rating resulted in a voltage of 33 V. Using this value in circuit simulation resulted in a coil current of 9 μA and a voltage of 0.4 V at the preamplifier input (Fig. 6c). Measurements showed a voltage of 0.7 V at the preamplifier input, dropping further to 50 mV after the preamplifier DC block input capacitor.

The shifts in the resonance frequency of the coil array elements, measured when the TMS coil was placed on the top of the coil array, are shown in Figure 6d. The results indicate that the resonance frequencies were increased by the presence of the TMS. Shifts ranged from 0.4 to 5.6 MHz, depending on the position of the TMS coil with respect to the elements. These frequency shifts were compensated for by retuning the coil elements prior to use.

After validating the low impact of the interactions between the coil array and the TMS system, measurements were carried out in the MR scanner to show the achieved sensitivity with the new hardware coil combined with the TMS system.

### Performance Comparison to Conventional Setup

**Figure 7:**
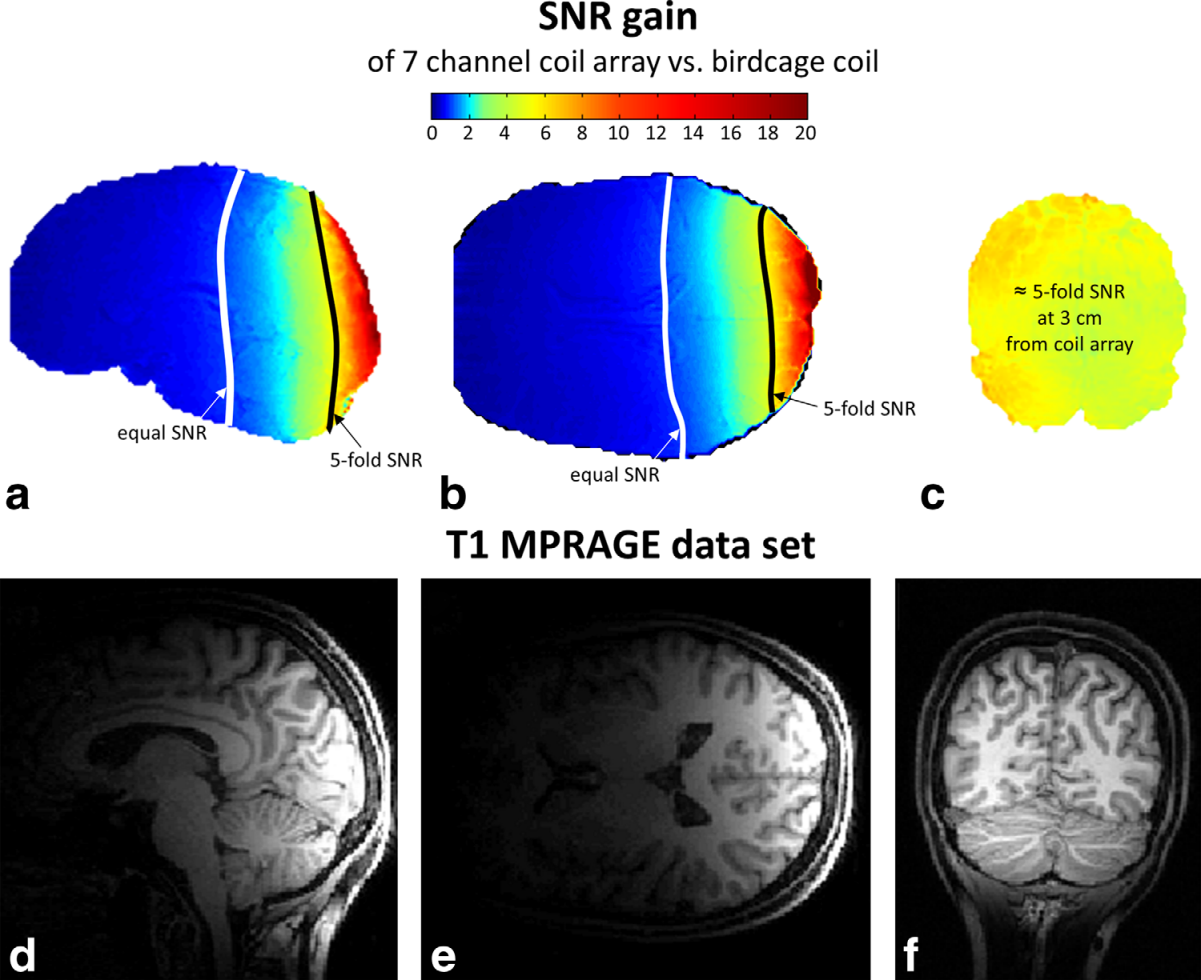
(a–c) SNR gain maps comparing the conventional setup and the new setup in vivo. SNR maps were derived based on 3D‐gradient echo data (TE/TR = 2.48 ms/7.92 ms, voxel size = 1 × 1 × 1 mm^3^). The lines of five‐fold SNR increase, and equal SNR of the coil array versus the birdcage coil are delineated. (d–f) In vivo images acquired with the new MR coil array for concurrent TMS/fMRI studies (T1‐MPRAGE sequence).

The results of the comparison of the new (coil array between head and TMS) and conventional (TMS between head and birdcage coil) setups are summarized at the top of Figure 7. The SNR gain as the ratio of the SNR maps of the new setup with respect to the conventional setup is shown in Figures 7a–c. The figure shows a representative slice for each slice orientation: a) sagittal, b) transversal, and c) coronal. The coronal slice was selected to show the results at a depth of 3 cm from the coil array, approximately at the depth of TMS stimulation. There, the SNR is improved by a factor of 5.1 on average. The coil array achieves an SNR better than or equal to the SNR of the birdcage coil in 42% of the whole brain volume.

Figures 7d–f present in vivo images acquired with the new hardware in combination with the TMS device.

### g‐Factor Maps

**Figure 8:**
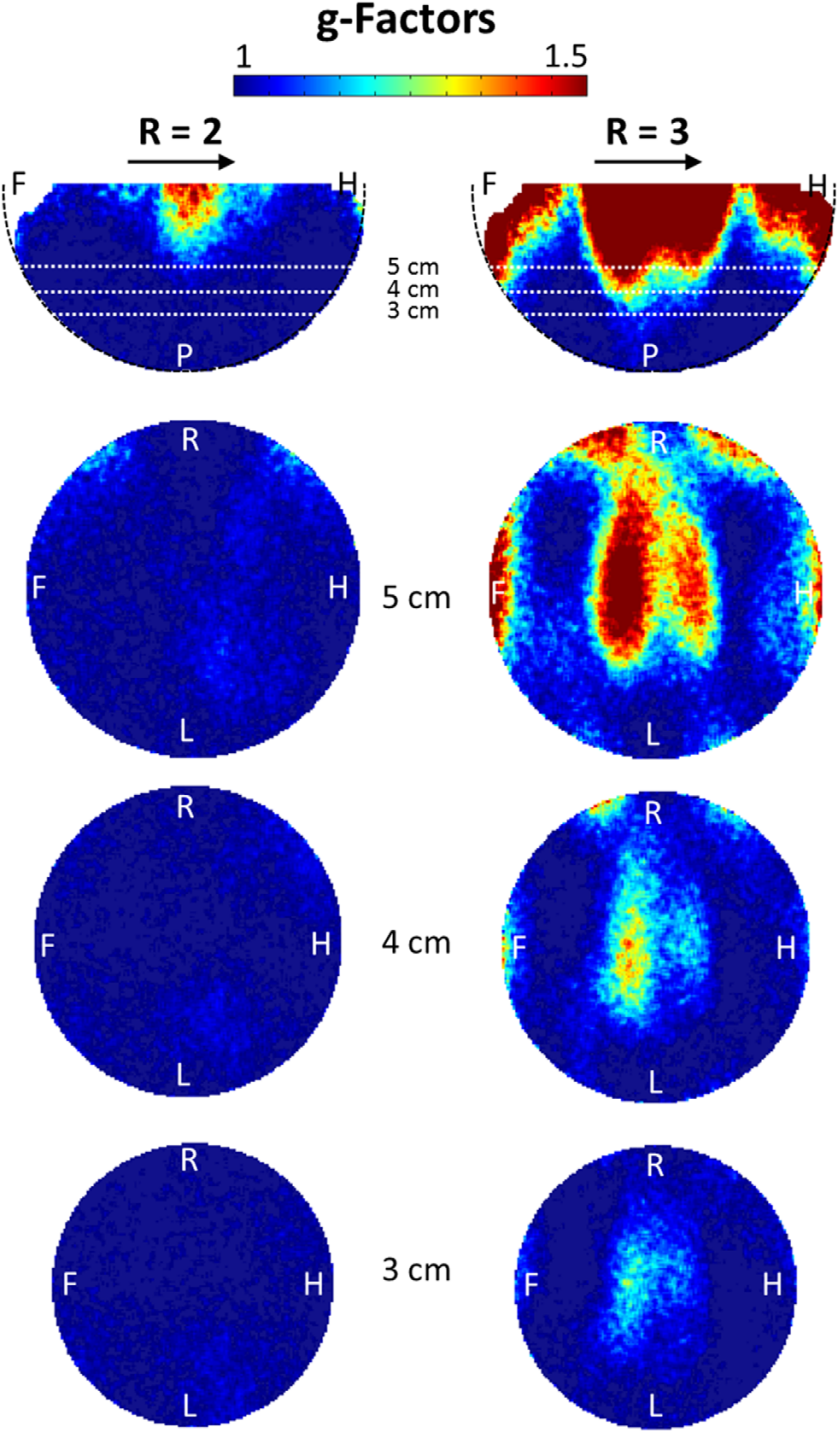
g‐Factor maps for different slices at acceleration factors R = 2 and R = 3. The direction of acceleration is indicated by the arrows. A, anterior; F, foot; H, head; L, left; P, posterior; R, right.

The g‐factor maps with acceleration factors R = 2 and R = 3 for different slices are shown in Figure 8. With R = 2, the g‐factor values were lower than 1.28 over all slices for depths up to 5 cm from the coil array, resulting in a worst‐case reduction of 1.81 in SNR in some regions as compared to the nonaccelerated case. For an acceleration factor of R = 3, the highest g‐factor value was 1.85.

### TMS/fMRI Experiment

An activation map superimposed onto the corresponding T1‐MPRAGE image is shown in Figure 4c. The activation maximum was found in the hand area of the primary motor cortex (*p* < 0.05; family wise error corrected). The time course of the peak activation voxel and modeled blood oxygen level‐dependent response of the experimental condition are shown in Figure 4b. The acquired EPI images (not shown) did not show any kind of image artifacts related to simultaneous rTMS stimulation and fMRI acquisition, confirming that the interactions between TMS and the MR coil array were negligable, and the time between TMS pulses and MR acquisition was sufficient.

## DISCUSSION

A dedicated 7‐channel receive‐only MR coil array for concurrent TMS/fMRI studies is presented. Due to its reduced size and improved handling, as compared to the state‐of‐the art setup employing a large birdcage coil, limitations in TMS positioning can be overcome because the TMS can now be placed freely.

In principle, it would be conceivable to build larger whole‐head coil arrays; however, their design would be limited by the fact that the TMS positioning system has to access the head from behind. Therefore, the classical conformal head array design, which is closed in that direction, is not applicable. A viable solution could be a large cylindrical array coil that is built approximately in the shape of a large birdcage coil. This in turn would again impose the positioning limitations given in the birdcage configuration. Additionally, the achievable SNR would be limited due to the larger distance between head and coil and the required larger size of the individual elements.

The measured and calculated voltages and currents induced by the TMS in the MR coil elements are very small; thus, they do not cause any relevant TMS field distortions or hardware damage. Additionally, the obtained results can be considered as worst‐case estimates because the loop coil was placed directly under the TMS coil at the position with the largest flux density, whereas the actual setup includes a distance of at least 5 mm between the coil elements and the TMS coil conductor. Furthermore, the central coil element, which is closest to the TMS coil, will not experience any significant induced voltage because the surface normal flux‐density component is effectively zero close to the center of a figure‐eight (TMS) coil.

The TMS efficiency loss of ≈ 15% corresponds to the expected field decay at 4.5 mm depth, which is equal to the thickness of the coil array at the focus point. This means that the TMS system and the MR coil array do not significantly interfere with each other. Consequently, the new setup can be considered safe for patients as well as for the hardware. For the average head shape, almost all areas of the brain are accessible to TMS stimulation with the coil array attached, as can be seen from Figure 3. However, because the coil array has a spherical concave shape, additional spacing between the head and the center of the coil might occur in areas where the curvature of the head is smaller than the curvature of the coil. This could impose practical restrictions for unusually large and/or flat head shapes.

In TMS experiments in humans, the achievable distance between the TMS coil and the stimulated cortical area is about 4 cm 33. Assuming that the TMS coil's housing is ≈ 1 cm thick, the cortical region stimulated by the TMS is typically located at a distance of 3 cm from the head surface. The presented experiments show that a five‐fold gain in SNR can be achieved in these areas with the new setup. The fraction of the brain where higher or equal SNR could be achieved using the presented array coil was 42%. To overcome this limitation, for example, when brain areas far from the point of TMS stimulation are of importance, or for studying whole‐brain network effects of TMS, an additional identical MR coil array could be positioned on the contralateral side of the head, allowing for improved SNR in most areas of the brain.

The noise correlation matrix showed that coupling between the array elements was low and dominated by intrinsic noise correlation due to overlapping sensitivity profiles (12% for neighboring elements vs. 8% for non‐neighboring elements). This results in moderate g‐factors; therefore, when using the new coil array, parallel imaging with an acceleration factor of 2 becomes feasible for TMS/fMRI experiments. The new coil setup can be readily combined with the latest advances in MR imaging such as multiband EPI and recent developments in TMS‐fMRI artifact compensation of B_0_ and leakage currents 33.

Foam padding in the form of a ring along the outer rim of the coil was used in the first in vivo experiments. Using such a ring, the distance of the target region to the center of the coil is not increased while ensuring acceptable comfort for the subject.

Improved fMRI data quality in terms of sensitivity gain and shorter acquisition times for these challenging experiments can help understanding complex brain physiology and explain, for example, the efficacy of revolutionary therapeutic methods using TMS—especially for major depression 9, 10, 56, 57, 58, 59, 60, which would give a better understanding of this common pathology.

## CONCLUSION

In conclusion, this novel coil array has proven to be safe, strongly improves the SNR in concurrent TMS/fMRI experiments, enables parallel imaging, and allows for flexible positioning of the TMS on the head while ensuring efficient TMS stimulation due to the ultraslim design.
